# Protective effect of L-carnitine on myocardial injury in rats with heatstroke^1^

**DOI:** 10.1590/ACB351206

**Published:** 2021-01-20

**Authors:** Xisong Wang, Yingyu Liu, Chunlai Zhang, Qing Song

**Affiliations:** I PhD, Department of Critical Care Medicine, Chinese People’s Liberation Army General Hospital, People’s Liberation Army Medical College, Beijing, China.; II Master, Department of Respiratory Medicine, Tangshan Central Hospital, Tangshan, China.; III Master, Department of Cardiology, Tangshan Central Hospital, Tangshan, China.; IV Master, Department of Critical Care Medicine, Chinese People’s Liberation Army General Hospital, People’s Liberation Army Medical College, Beijing, China.

**Keywords:** Carnitine, Heatstroke, Myocardial Reperfusion Injury, Rats

## Abstract

**Purpose:**

To investigate the protective effect of L-carnitine on myocardial injury in rats with heatstroke.

**Methods:**

orty-eight rats were randomly divided into control, heatstroke and 25, 50 and 100 mg/kg L-carnitine groups. The last three groups were treated with 25, 50 and 100 mg/kg L-carnitine, respectively, for seven successive days. Then, except for the control group, the other four groups were transferred into the environment with ambient temperature of (39.5 ± 0.4 °C) and relative humidity of (13.5 ± 2.1%) for 2 h. The core temperature (Tc), mean arterial pressure (MAP), heart rate (HR) and serum and myocardial indexes were detected.

**Results:**

Compared with the heatstroke group, in the 100 mg/kg L-carnitine group, the Tc was significantly decreased, the MAP and HR were significantly increased, the serum creatine kinase, lactate dehydrogenase, alkaline phosphatase, aspartate aminotransferase, tumor necrosis factor α and interleukin 1β levels were significantly decreased, the myocardial superoxide dismutase and glutathione peroxidase levels were significantly increased, the myocardial malondialdehyde level was significantly decreased and the cardiomyocyte apoptosis index and myocardial caspase-3 protein expression level were remarkably decreased (p < 0.05).

**Conclusions:**

The L-carnitine pretreatment can alleviate the myocardial injury in heatstroke rats through reducing the inflammatory response, oxidative stress and cardiomyocyte apoptosis.

## Introduction

Heatstroke is a serious manifestation of heat injury and a serious life-threatening disease. Clinically, it is defined as the core temperature (T_c_) being over 40 °C, body temperature regulation failure and central nervous system symptoms, such as convulsion, coma, delirium and others^1^^,^ 2. With the global warming, the incidence of heatstrokes is increasing year by year. The heatstroke can cause the systemic inflammatory response, which leads to multiple organ dysfunction or failure, including hemorrhage and necrosis in the brain, lung, heart, gastrointestinal tract and kidney^2^. It is reported that the heat stress can induce the cardiomyocyte apoptosis and heart dysfunction^3^. L-carnitine is a compound with multiple physiological functions, which mainly exists in the heart and skeletal muscle of the body^4^. On one hand, as the carrier of fatty acid transportation, L-carnitine can transfer the medium and long-chain fatty acids from the outside of cell mitochondrial membrane to the inside of membrane, which are then oxidized in the mitochondrial matrix for generating energy^5^. On the other hand, L-carnitine is an antioxidant with the function of scavenging free radicals. It can capture the free radicals and promote the reacylation of membrane phospholipids, which is conducive to the repair of biofilm and resistance of oxidation^6^^,^ 7. In this study, the heatstroke model of rats was established, and the protective effect and mechanism of L-carnitine on myocardial injury in rats with heatstroke were investigated.

## Methods

This study was performed with the approval of the ethics committee of Chinese PLA General Hospital, PLA Medical College. All animal procedures followed the Guide for the Care and Use of Laboratory Animals by the National Institutes of Health.

Forty-eight male Sprague-Dawley rats (220–250 g) were adaptively fed in normal condition for seven days. Then, the rats were randomly divided into control group, heatstroke group and 25, 50 and 100 mg/kg L-carnitine groups, with 8 rats in each group. The last three groups were treated with 25, 50 and 100 mg/kg L-carnitine by intraperitoneal injection, respectively. The treatment was conducted once per day, for seven successive days. The control and heatstroke groups were synchronously given with same volume of normal saline.

### Modeling of heatstroke

On the eighth day of the experiment, except for the control group, the other four groups were transferred to the artificial climate chamber with ambient temperature of (39.2 ± 0.4 °C) and relative humidity of (13.5 ± 2.1%), with water deprivation and fasting. The modeling was performed for 2 h. During the modeling, no rat died in any group.

### Measurement of T_c_, mean arterial pressure and heart rate of rats

After 2h of modeling, the rats were anesthetized. The femoral artery was separated, followed by intubation and connection to the multichannel physiological recorder through pressure transducer. The T_c_, mean arterial pressure (MAP) and heart rate (HR) were measured.

### Detection of serum indexes

The rats were anesthetized by intraperitoneal injection of 3% sodium pentobarbital and then were executed by decapitation. The vena cava blood was taken. After centrifuged at 3000 rpm and 4 °C for 15 min, the serum was obtained. The serum creatine kinase (CK), lactate dehydrogenase (LDH), alkaline phosphatase (ALP) and aspartate aminotransferase (AST) levels were detected by automatic biochemical detector. The serum tumor necrosis factor α (TNF-α) and interleukin 1ß (IL-1ß) were detected by enzyme linked immunosorbent assay.

### Determination of myocardial oxidative stress indexes

The heart tissue of rats was taken and homogenized with normal saline under the condition of ice bath. After centrifuged at 3000 rpm and 4 °C for 15 min, the supernatant was obtained. The levels of superoxide dismutase (SOD), glutathione peroxidase (GSH-Px) and malondialdehyde (MDA) were determination by ultraviolet-visible spectrophotometry.

### Determination of cardiomyocyte apoptosis indexes

The heart tissue of rats was taken. The cardiomyocyte apoptosis was determined using the TUNEL method and the apoptotic index (percentage of apoptotic cells in total cells) was calculated^8^. In addition, the expression level of Caspase-3 protein in myocardial tissue was determined by western blotting^9^. The determination procedures were according to the instructions of kits.

### Statistical analysis

Group data were presented as mean ± standard deviation. The differences among the experimental groups were examined using one-way analysis of variance (ANOVA) with post-hoc Tukey test. P < 0.05 was considered statistically significant.

## Results

### Effect of L-carnitine on T_c_, MAP and HR in thermal injury rats

**Figure 1:**
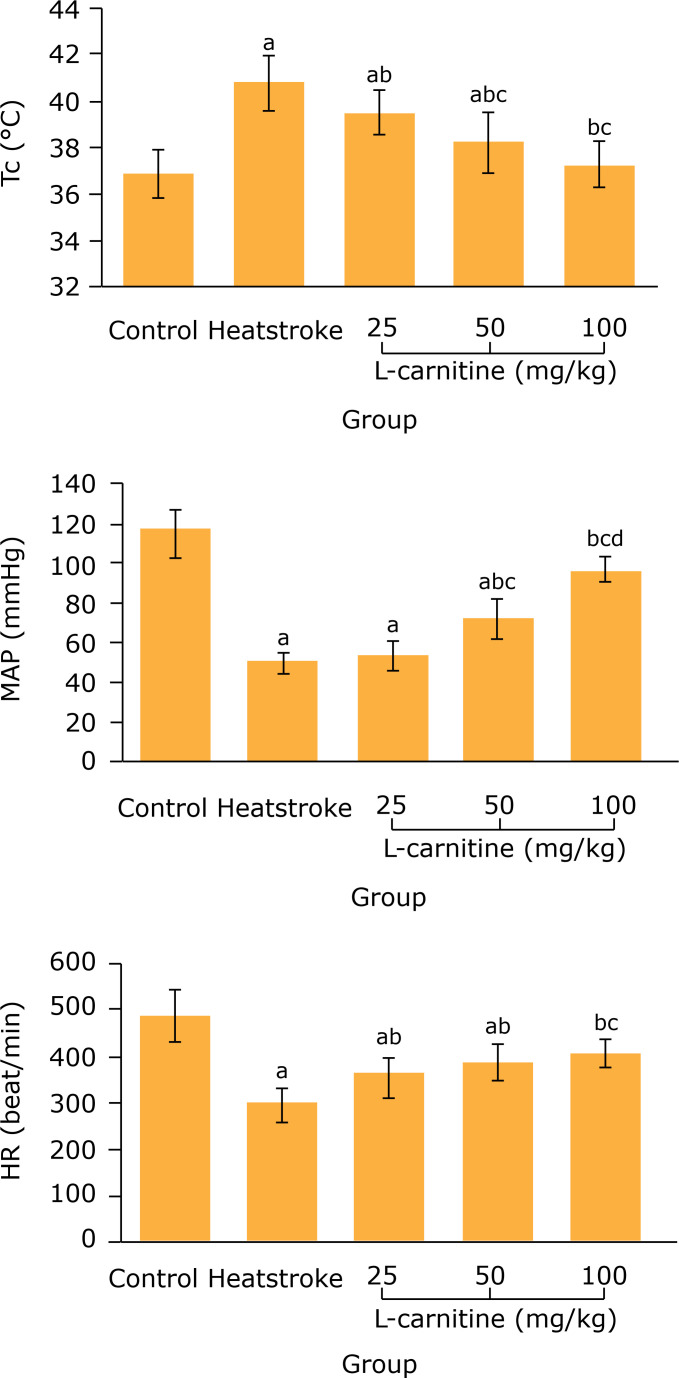
Comparison of T_c_, map and hr among five groups (n = 8). ^a^P < 0.05 *vs.* control group; ^b^P < 0.05 *vs*. heatstroke group; ^c^P < 0.05 *vs.* 25 mg/kg L-carnitine group; ^d^P < 0.05 *vs.* 50 mg/kg L-carnitine group. T_c_: core temperature; MAP: mean arterial pressure; HR: heart rate.

After 2 h of heatstroke modeling, in the heatstroke group, the T_c_ significantly increased (p < 0.05), and the MAP and HR significantly decreased, (p < 0.05) compared with the control group. Compared with heatstroke group, the T_c_ in 25, 50 and 100 mg/kg L-carnitine groups significantly decreased (p < 0.05) and the MAP in 50 and 100 mg/kg L-carnitine groups and HR in 25, 50 and 100 mg/kg L-carnitine groups significantly increased (p < 0.05) (Fig. 1).

### Effect of L-carnitine on serum CK, LDH, ALP and AST levels in thermal injury rats

**Figure 2:**
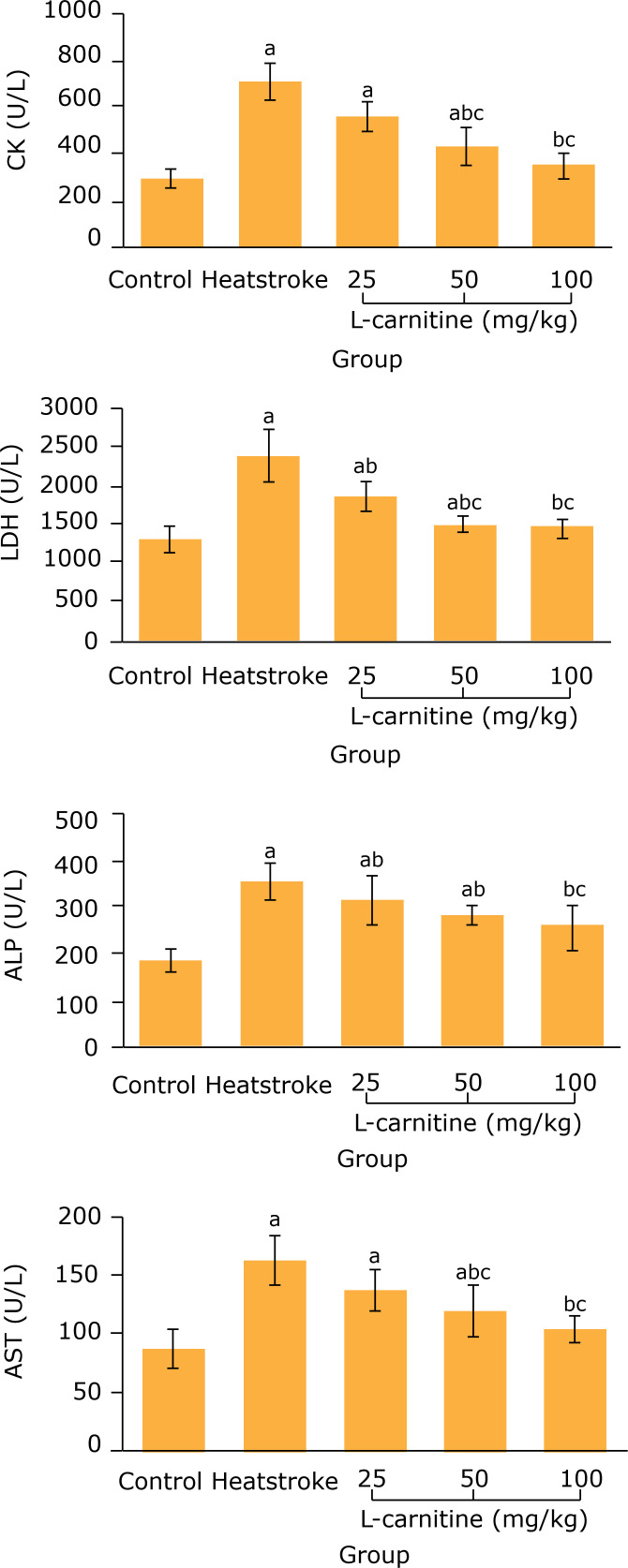
Comparison of serum CK, LDH, ALP and AST levels among five groups (n = 8). **^a^**P < 0.05 vs. control group; **^b^** P < 0.05 *vs*. heatstroke group; **^c^** P < 0.05 *vs*. 25 mg/kgL-carnitine group; ^d^P < 0.05 *vs*. 50 mg/kg L-carnitine group. CK: creatine kinase; LDH: lactate dehydrogenase; ALP: alkaline phosphatase; AST: aspartate aminotransferase.

As shown in Fig. 2, after 2 h of heatstroke modeling, the serum CK, LDH, ALP and AST levels in heatstroke group weresignificantly higher than those in the control group (p < 0.05).

Compared with the heatstroke group, the serum CK and AST levels in 50 and 100 mg/kg L-carnitine groups and the serum LDH and ALP levels in 25, 50 and 100 mg/kg L-carnitine groups significantly decreased (p < 0.05).

### Effect of L-carnitine on serum TNF-α and IL-1β levels in thermal injury rats

**Figure 3:**
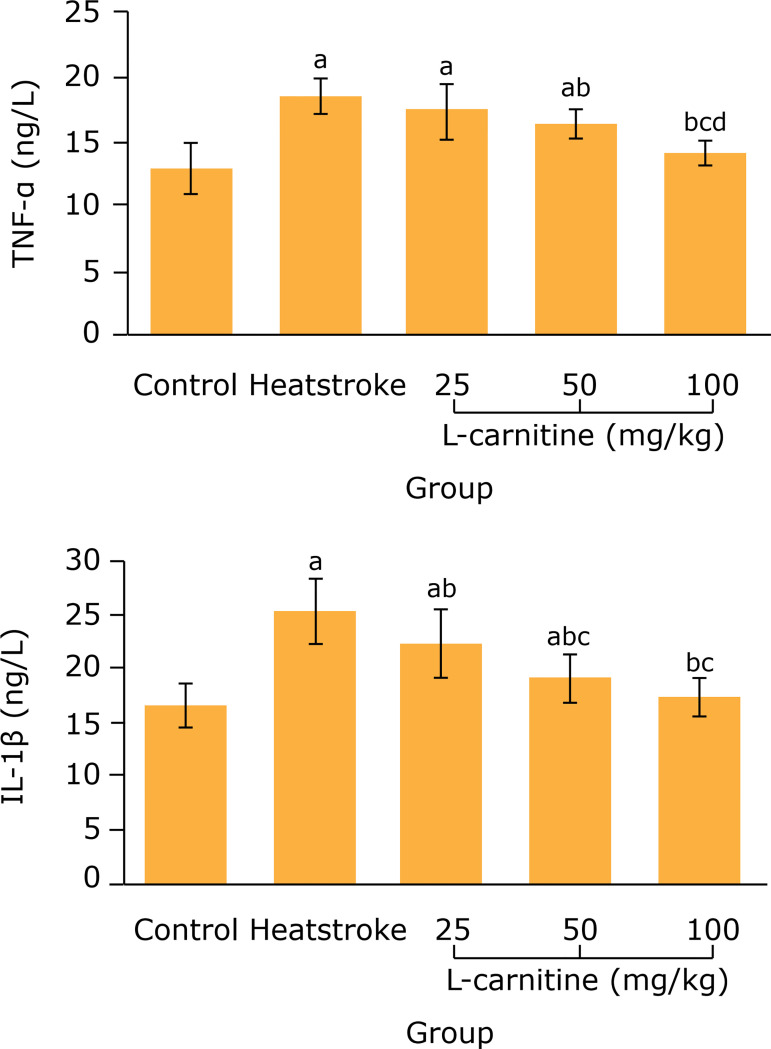
Comparison of serum TNF-α and IL-1β levels among five groups (n = 8). ^a^P < 0.05 *vs.* control group; ^b^P < 0.05 *vs.* heatstroke group; ^c^P < 0.05 *vs.* 25 mg/kgL-carnitine group; ^d^P < 0.05 *vs.* 50 mg/kg L-carnitine group. TNF-α: tumor necrosis factor α; IL-1β: interleukin 1β.

Figure 3 showed that, compared with the control group, the serum TNF-α and IL-1β levels in the heatstroke group were remarkably increased (p < 0.05). Compared with the heatstroke group, the serum TNF-α level in 50 and 100 mg/kg L-carnitine groups and the IL-1β level in 25, 50 and 100 mg/kg L-carnitine groups were remarkably decreased (P < 0.05).

### Effect of L-carnitine on myocardial SOD, GSH-Px and MDA levels in thermal injury rats

**Figure 4:**
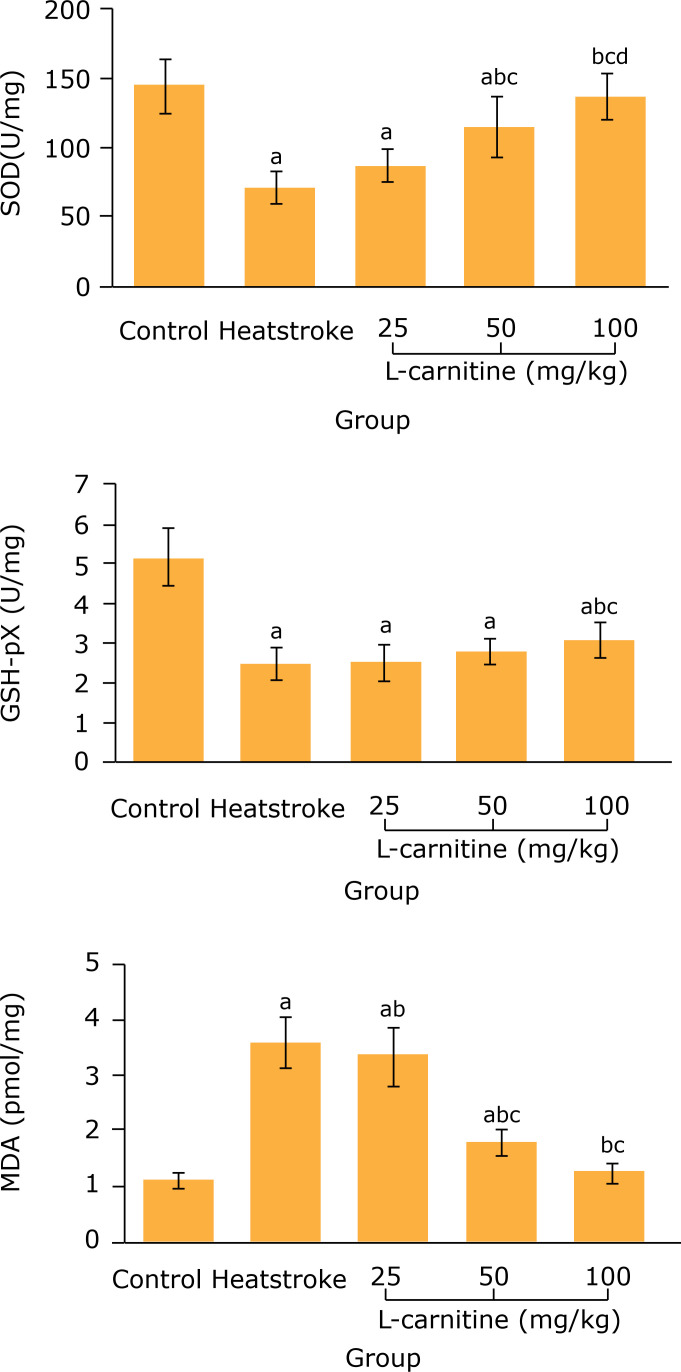
Comparison of myocardial SOD, GSH-Px and MDA levels among five groups (n = 8). ^a^P < 0.05 *vs.* control group; ^b^P < 0.05 *vs*. heatstroke group; ^c^P < 0.05 *vs.* 25 mg/kg L-carnitine group; ^d^P < 0.05 *vs.* 50 mg/kg L-carnitine group. SOD: superoxide dismutase; GSH-Px: glutathione peroxidase; MDA: malondialdehyde.

After 2 h of heatstroke modeling, the myocardial SOD and GSH-Px levels in the heatstroke group significantly decreased, compared with the control group (p < 0.05), and the myocardial MDA level significantly increased(p < 0.05). Compared with the heatstroke group, the myocardial SOD level in 50 and 100 mg/kg L-carnitine groups and GSH-Px level in 100 mg/kg L-carnitine group significantly increased (p < 0.05), and the myocardial MDA level in 25, 50 and 100 mg/kg L-carnitine groups significantly decreased (p < 0.05) (Fig. 4).

*Effect of L-carnitine on cardiomyocyte apoptosis index and myocardial caspase-3 protein expression in thermal injury rats*

**Figure 5:**
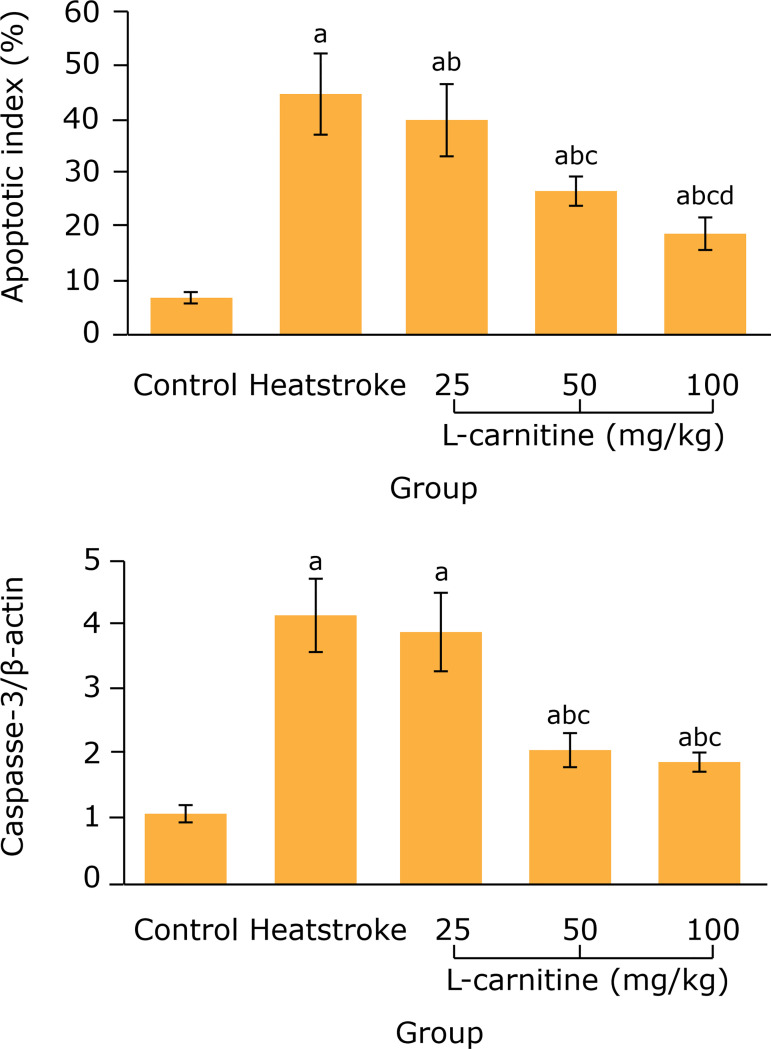
Comparison of cardiomyocyte apoptosis index and myocardial caspase-3 protein expression among five groups (n = 8). ^a^P < 0.05 *vs*. control group; ^b^P < 0.05 vs. heatstroke group; ^c^P < 0.05 *vs.* 25 mg/kg L-carnitine group; ^d^P < 0.05 *vs.* 50 mg/kg L-carnitine group.

Compared with the control group, the cardiomyocyte apoptosis index and myocardial caspase-3 protein expression levels in the heatstroke group were remarkably increased (p < 0.05). Compared with the heatstroke group, the cardiomyocyte apoptosis index in 25, 50 and 100 mg/kg L-carnitine groups and the myocardial caspase-3 protein expression level in 50 and 100 mg/kg L-carnitine groups were remarkably decreased (p < 0.05) (Fig. 5).

## Discussion

This study has established the heatstroke model of rats and investigated the protective effect of L-carnitine on myocardial injury in heatstroke rats. The changes of T_c_, MAP and HR are the important indications of heatstroke. Results of this study showed that, after 2 h of modeling, the T_c_ significantly decreased and the MAP and HR significantly increased in 50 and 100 mg/kg L-carnitine groups, when compared with the heatstroke group. This indicates that the L-carnitine pretreatment can lighten the symptoms of heatstroke in rats. Creatine kinase, LDH, ALP and AST widely exist in the cytoplasm and mitochondria of tissue cells of myocardium and skeletal muscle. When the myocardium is damaged, the levels of these enzymes show different degrees of elevation. They are released into the blood in large quantities. Therefore, the serum CK, LDH, ALP and AST levels can reflect the degree of myocardial injury^10^^,^ 11. Results of this study showed that the serum CK, LDH, ALP and AST levels in 50 and 100 mg/kg L-carnitine groups significantly decreased compared with the heatstroke group. This suggests that the L-carnitine pretreatment can alleviate the myocardial injury in heatstroke rats.

Heatstroke can cause the systemic inflammatory response syndrome^12^. The cytokines, such as TNF-α and IL-1β, are closely related to the occurrence and development of systemic inflammatory response syndrome. Tumor necrosis factor α is a key factor in inflammatory response and plays an important role in neutrophil recruitment and inflammatory cascade reaction. In addition, TNF-α can induce the production of other inflammatory cytokines, such as IL-1β and IL-6, which can also stimulate the migration and adhesion of neutrophils and induce the inflammatory cascade reaction, thus leading to the tissue damage^13^. The increased serum TNF-α and IL-1β levels are closely related to the myocardial injury^14^. In the present study, the serum TNF-α and IL-1β levels in 50 and 100 mg/kg L-carnitine groups were remarkably decreased compared with heatstroke group. This suggests that, the L-carnitine pretreatment can reduce the inflammatory response, which may be related to its alleviation of myocardial injury in heatstroke rats.

Oxidative stress of cells can cause the release of toxic free radicals from endothelial cells and vascular smooth muscle cells. It is found that the oxidative stress plays an important role in the pathogenesis of cardiovascular disease, including atherosclerosis, hypertension, vascular endothelial dysfunction and ischemic heart disease^15^. Superoxide dismutase and GSH-Px are the main antioxidant enzymes in the body. Malondialdehyde is the product of lipid peroxidation. Therefore, the myocardial SOD, GSH-Px and MDA levels can be used as indirect indicators of myocardial oxidative stress injury^16^. Results of this study showed that the myocardial SOD and GSH-Px levels were significantly increased and the myocardial MDA level was significantly decreased in the 100 mg/kg L-carnitine group, when compared with the heatstroke group. It can be concluded that, the L-carnitine can resist the myocardial oxidative stress, thus alleviating the myocardial injury in heatstroke rats.

Apoptosis is a process of programmed active death of cells under certain physiological or pathological conditions for maintaining the stability of their internal environment. Heatstroke is often manifested as systemic inflammatory response syndrome or sepsis. In heatstroke, the increaseof inflammatory cytokines in body leads to the aggregation ofneutrophils and other leukocytes, which release toxins to induce the cell apoptosis^17^. Caspases are the promoters and executors of apoptosis in mammalian cells^18^. Caspase-3 is the most important apoptotic protease in the downstream of Caspases cascade. It is also a marker enzyme of cell apoptosis^19^. In this study, the cardiomyocyte apoptosis index and myocardial caspase-3 protein expression level were remarkably decreased in the 50 and 100 mg/kg L-carnitine groups, compared with the heatstroke group. This indicates that, L-carnitine can reducethe cardiomyocyte apoptosis, which contributes to the alleviation of myocardial injury in heatstroke rats.

## Conclusions

It has been firstly demonstrated that the L-carnitine pretreatment can alleviate the myocardial injury in heatstroke rats through reducing inflammatory response, oxidative stress and cardiomyocyte apoptosis. This study has some limitations. Firstly, the heatstroke can cause the systemic inflammatory response syndrome. The changes of serum CK, LDH, ALP and AST levels are not the specific markers of myocardial injury. They may be affected by the injury of other organs. Therefore, in a next study, the changes of serum CK, LDH, ALP and AST levels to reflect the myocardial injury should be further investigated under the condition that other organs are also affected by heatstroke. Secondly, there may be other mechanisms for the protective effect of L-carnitine on myocardial injury in heatstroke, which needs to be confirmed by more studies.
